# Fixing imbalanced binary classification: An asymmetric Bayesian learning approach

**DOI:** 10.1371/journal.pone.0311246

**Published:** 2024-10-16

**Authors:** Letícia F. M. Reis, Diego C. Nascimento, Paulo H. Ferreira, Francisco Louzada

**Affiliations:** 1 Institute of Mathematics and Computer Sciences, University of São Paulo, São Carlos, São Paulo, Brazil; 2 Department of Mathematics, University of Atacama, Copiapó, Atacama, Chile; 3 Department of Statistics, Federal University of Bahia, Salvador, Bahia, Brazil; Buckinghamshire New University - High Wycombe Campus: Buckinghamshire New University, UNITED KINGDOM OF GREAT BRITAIN AND NORTHERN IRELAND

## Abstract

Most statistical and machine learning models used for binary data modeling and classification assume that the data are balanced. However, this assumption can lead to poor predictive performance and bias in parameter estimation when there is an imbalance in the data due to the threshold election for the binary classification. To address this challenge, several authors suggest using asymmetric link functions in binary regression, instead of the traditional symmetric functions such as logit or probit, aiming to highlight characteristics that would help the classification task. Therefore, this study aims to introduce new classification functions based on the Lomax distribution (and its variations; including power and reverse versions). The proposed Bayesian functions have proven asymmetry and were implemented in a Stan program into the R workflow. Additionally, these functions showed promising results in real-world data applications, outperforming classical link functions in terms of metrics. For instance, in the first example, comparing the reverse power double Lomax (RPDLomax) with the logit link showed that, regardless of the data imbalance, the RPDLomax model assigns effectively lower mean posterior predictive probabilities to failure and higher probabilities to success (21.4% and 63.7%, respectively), unlike Logistic regression, which does not clearly distinguish between the mean posterior predictive probabilities for these two classes (36.0% and 39.5% for failure and success, respectively). That is, the proposed asymmetric Lomax approach is a competitive model for differentiating binary data classification in imbalanced tasks against the Logistic approach.

## 1 Introduction

Various classification tasks are subconsciously undertaken on a daily basis. Clothes are sorted and placed in appropriate drawers, messages and emails are prioritized, dishes are categorized as either dirty or clean, and tasks are assessed based on difficulty levels. While seemingly straightforward, these activities do not entail critical consequences when missclassified. Conversely, the failure of a medical professional to diagnose cancer could lead to the patient’s demise in a short period. Similarly, frequent misclassification of clients as either good or bad payers by a financial institution may result in significant financial losses, potentially reaching billions. The significance of accurate modeling and classification becomes apparent in scenarios where high stakes underscore their pivotal role across diverse domains.

The term “imbalanced binary data” denotes a dataset in which one of the classes significantly outweighs the other in terms of observations. For example, in default prediction, there are typically more good payers than bad payers, and in cancer detection, the number of healthy individuals far exceeds those diagnosed with the disease. This imbalance poses as a challenge for binary data modeling and classification, as most machine learning algorithms and statistical models presume an even distribution of observations across both categories [1]. Consequently, these algorithms often prioritize the majority class over the minority class, even though the minority class is frequently of greater interest.

In binary regression, symmetric link functions, such as logit and probit functions, are often chosen, implying equal probabilities for both categories. However, recent research, exemplified by [2], argues that asymmetric link functions may better suit the task of handling imbalanced data by allowing for distinct probabilities for each category. Adopting symmetric link functions in such cases, as noted by [3], can result in substantial bias in parameter estimation and success probability prediction.

To address this challenge, [4] proposed a method to transform well-known distributions to derive more flexible and asymmetric link functions. This approach involves exponentiating existing cumulative distribution functions by the positive parameter λ, acting as an asymmetry parameter, providing control over the distribution’s shape. This transformation introduces asymmetry and maintains a connection with symmetric link functions.

The main objective of this paper is to introduce novel asymmetric classification functions based on the Lomax distribution to improve the modeling and classification of imbalanced binary data. The proposed models offer the advantage of incorporating only one additional parameter, thereby establishing a parametrically defined alternative to traditional binary regression methods. This approach ensures interpretability, leveraging the asymmetry generation method proposed by [4].

### 1.1 Contributions

The main contributions of the proposed models are defined as follows:

i) Propose new asymmetric classification functions that outperform traditional symmetric functions in classifying imbalanced data.
ii) Compare the proposed link functions with traditional link functions (logit, probit, cauchit, loglog, and cloglog), focusing on the logit link due to its popularity in classification.
iii) Introduce new asymmetric functions that require only one additional parameter to generate asymmetry. This approach reduces variance in parameter estimation and enhances model stability, offering a more robust alternative to other asymmetric classification functions.
iv) Provide a model that can be easily implemented within the R workflow, facilitating its adoption and application in real-world data analysis.
v) Ensure the parametric models are interpretable, with all parameters clearly defined. The inclusion of the asymmetry parameter λ allows for a direct relationship with symmetric classification models, regulating the model’s asymmetry.
vi) Present a Bayesian approach to interpret the models, offering a comprehensive understanding of the parameter distributions and their impact on classification.

### 1.2 Article organization

The structure of this article is as follows. In Section 2, related works in literature are discussed. Section 3 introduces the asymmetric Lomax distributions. Section 4 presents the Bayesian binary regression model using the distributions introduced in Section 3. Section 5 provides the results of simulations and Bayesian analysis of these distributions. In Section 6, the proposed methodology is exemplified in two applications: one related to image classification (wilt), and another related to blood donation. Section 7 discusses the results of this work. Finally, Section 8 contains some final remarks and future research directions.

## 2 Literature review

In academic literature, several authors have explored asymmetric link functions as alternatives to conventional symmetric models. Notable examples include [5], who explored the performance of various asymmetric link functions in predicting mortality in life insurance, and [6], who employed the asymmetric Student’s t-distribution to identify patients with Parkinson’s disease. Additionally, [7] investigated Fréchet, Weibull, and Gumbel link functions to model bankruptcy occurrences in small and medium-sized businesses. Nevertheless, it is crucial to acknowledge that these models often lack a mechanism for controlling asymmetry through an additional parameter, making it challenging to establish connections with conventional symmetric models [2].

In addition to these models, researchers have made efforts to transform or generalize well-known distributions to obtain more flexible and asymmetric link functions in binary regression. Noteworthy examples include the asymmetric logistic distribution addressed by [8], and the exponentiated-exponential logistic distribution explored by [9]. Furthermore, it is worth mentioning the logisticF and logisticKZ distributions presented by [10], as well as the generalization proposed by [11] based on the log F family, encompassing a range of models. A common feature of these models is the introduction of at least two additional parameters, raising concerns about the associated cost in terms of increased variance, as pointed out by [12].

In contrast, the approach presented by [4] offers a advantage as it requires the inclusion of just a single parameter, which in turn establishes a direct link with symmetric link functions. Despite its potential benefits, the use of this transformation in binary regression has been relatively underexplored. While it has been applied to classic models such as the normal, logistic, Cauchy, Student’s t, Laplace, and Gumbel distributions [4, 13–16], there remains ample opportunity to investigate its impact on a wider range of distributions.

To address this gap, our study aims to develop a new model by applying [4]’s transformation to the Lomax distribution. This paper explores Bazán’s method within this context, offering a novel approach to modeling and classifying imbalanced binary data with minimal additional parameters while maintaining interpretability.

## 3 Asymmetric Lomax distributions

In this section, we will introduce asymmetric Lomax models, namely the power double Lomax (PDLomax) and the reverse power double Lomax (RPDLomax) models. First, we will provide the definition of the double Lomax (DLomax) distribution proposed by [17], which is an extension of the Lomax distribution (also known as Pareto Type II distribution) on the real line. For further details on the Lomax distribution, see, e.g., [18, 19]. A random variable X∈R follows a DLomax distribution with parameters μ∈R and *σ* > 0 if its probability density function (pdf) and cumulative distribution function (cdf) are given, respectively, by

$$\begin{array}{c} g\left(x\right)=\frac{1}{2\sigma {\left(1+|\frac{x-\mu }{\sigma }|\right)}^{2}}\;\;\text{and}\;\;G\left(x\right)=\{\begin{array}{cc} \frac{1}{2(1+\frac{\mu -x}{\sigma })}, & x\leq \mu ,\\ 1-\frac{1}{2(1+\frac{x-\mu }{\sigma })}, & x>\mu . \end{array} \end{array}$$

If *μ* = 0 and *σ* = 1, then it is referred to as the standard DLomax distribution. [17] derived this distribution from the ratio of two independent and identically distributed standard classical Laplace random variables.

To construct new asymmetric link functions using the standard DLomax distribution as a base, we consider the power transformation proposed by [4]. Thus, if *F*_P_ is a power distribution with base distribution *G*, then its pdf and cdf are described by the following exponentiation process:

$$\begin{array}{c} f_{\text{P}}\left(x|\lambda \right)=\lambda g(x){\left[G(x)\right]}^{\lambda -1}\;\;\text{and}\;\;F_{\text{P}}\left(x|\lambda \right)={\left[G(x)\right]}^{\lambda }, \end{array}$$

with λ > 0. Now, *F*_RP_ is a reverse power distribution with base distribution *G*, and its pdf and cdf are described as follows:

$$\begin{array}{c} f_{\text{RP}}\left(x|\lambda \right)=\lambda g(-x){\left[G(-x)\right]}^{\lambda -1}\;\;\text{and}\;\;F_{\text{RP}}\left(x|\lambda \right)=1-{\left[G(-x)\right]}^{\lambda }, \end{array}$$

where λ > 0. For several properties that establish the relationship between the power and reverse power distributions, see, e.g., the work of [15].

Consequently, a random variable *X* has a standard PDLomax distribution with an asymmetry parameter λ > 0, if its pdf can be written as follows:

$$\begin{array}{c} f_{\text{P}}\left(x\right)=\{\begin{array}{cc} \frac{\lambda }{2{\left(1+|x|\right)}^{2}}{\left[\frac{1}{2(1-x)}\right]}^{\lambda -1}, & x\leq 0,\\ \frac{\lambda }{2{\left(1+|x|\right)}^{2}}{\left[1-\frac{1}{2(1+x)}\right]}^{\lambda -1}, & x>0, \end{array} \end{array}$$

with cdf given by

$$\begin{array}{c} F_{\text{P}}\left(x\right)=\{\begin{array}{cc} {\left[\frac{1}{2(1-x)}\right]}^{\lambda }, & x\leq 0,\\ {\left[1-\frac{1}{2(1+x)}\right]}^{\lambda }, & x>0. \end{array} \end{array}$$

Furthermore, a random variable *X* follows a standard RPDLomax distribution with an asymmetry parameter λ > 0, if its pdf is described as follows:

$$\begin{array}{c} f_{\text{RP}}\left(x\right)=\{\begin{array}{cc} \frac{\lambda }{2{\left(1+|x|\right)}^{2}}{\left[1-\frac{1}{2(1-x)}\right]}^{\lambda -1}, & x\leq 0,\\ \frac{\lambda }{2{\left(1+|x|\right)}^{2}}{\left[\frac{1}{2(1+x)}\right]}^{\lambda -1}, & x>0, \end{array} \end{array}$$

with cdf given by

$$\begin{array}{c} F_{\text{RP}}\left(x\right)=\{\begin{array}{cc} 1-{\left[1-\frac{1}{2(1-x)}\right]}^{\lambda }, & x\leq 0,\\ 1-{\left[\frac{1}{2(1+x)}\right]}^{\lambda }, & x>0. \end{array} \end{array}$$

Note that *F*_RP_(*x*) = 1 − *F*_P_(*x*). Thus, the PDLomax and RPDLomax distributions are distinct but closely related since one reflects the other. Also, *F*_P_(−*x*) ≠ 1 − *F*_P_(*x*) and *F*_RP_(−*x*) ≠ 1 − *F*_RP_(*x*), which shows that *F*_P_ and *F*_RP_ are not symmetric.

To the best of our knowledge, these two probability distributions have not been presented in the literature. However, their complete characterization (statistical properties such as moments, including the mean, variance, skewness and kurtosis) is not within the scope of this paper and will be discussed in the future.

**Fig 1:**
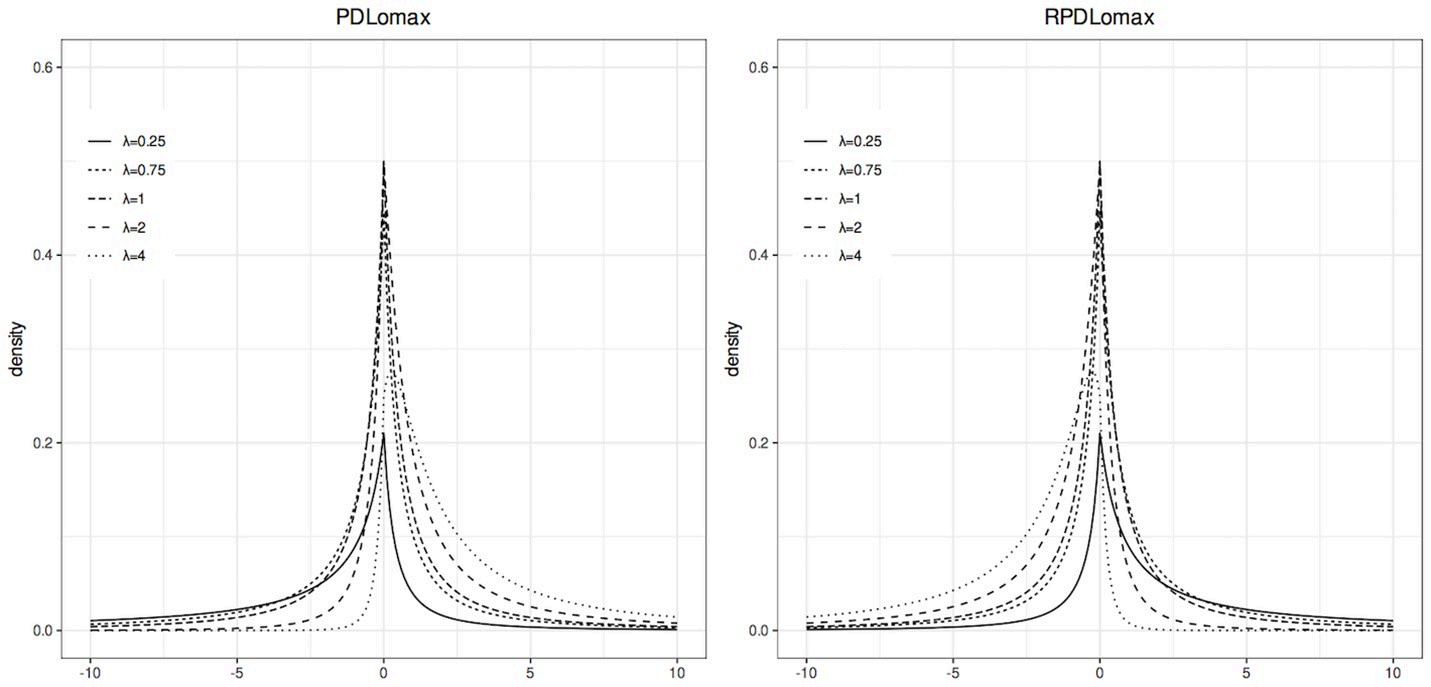
Probability density functions of the standard PDLomax and RPDLomax distributions at various values of λ.

**Fig 2:**
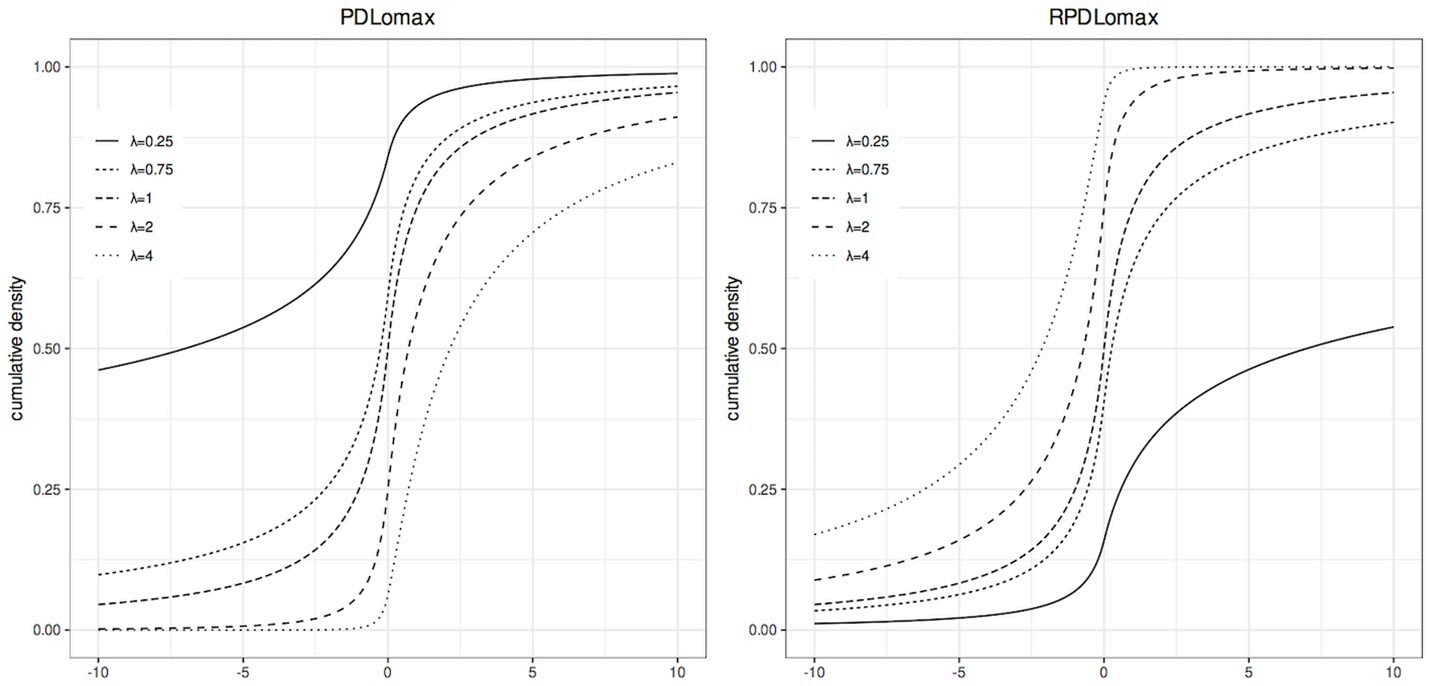
Cumulative distribution functions of the standard PDLomax and RPDLomax distributions at various values of λ.

Figs 1 and 2 show, respectively, the pdf and cdf plots of the standard PDLomax and RPDLomax distributions for various values of λ.

Fig 1 shows that the addition of the λ parameter can introduce both right (positive) and left (negative) skewness. In particular, for the standard PDLomax distribution (left panel), when λ < 1, the density curve concentrates to the left; when λ > 1, the density curve concentrates to the right; and at λ = 1, we have the original distribution (standard DLomax distribution). The standard RPDLomax distribution (right panel) exhibits the opposite behavior, as both functions are reflections of each other.

In Fig 2, one can see how the variation in λ modifies the cumulative density function. Note that the variation of this parameter not only affects the interval where the greatest probability is concentrated, but also affects the slope of the probability curve. In particular, for the standard PDLomax distribution (left panel), when λ < 1, it is more likely that *X* < 0, but when λ > 1, it is more likely that *X* > 0. That is, in the context of binary regression, in which this cdf is used as a link function, it is expected that when λ < 1, there is a greater proportion of failures than successes (more 0’s than 1’s); and when λ > 1, there must be a higher proportion of successes. When λ = 1, it is an atypical case in which successes and failures will be balanced. For the standard RPDLomax distribution (right panel), an inverse behavior is observed.

The λ parameter directly affects the rate at which the cdf increases. A larger λ results in a steeper cdf slope, making it approach 1 more quickly as *x* increases. Conversely, a smaller λ produces a gentler slope. In the RPDLomax distribution, a gentler slope (λ < 1) corresponds to a higher proportion of 0’s, enabling it to manage greater imbalances of 0’s more effectively than the PDLomax distribution. On the other hand, the PDLomax distribution exhibits gentler slopes (λ < 1) when there are more 1’s than 0’s, allowing it to handle higher proportions of 1’s.

## 4 Bayesian binary regression model

In this section, we will present the new asymmetric Lomax model for binary regression using the distributions introduced in the previous section. Utilizing the notation defined earlier, this model can be described by the following set of equations:

$$\begin{array}{c} Y_{i}|\beta ,\lambda \overset{\text{ind.}}{∼}\text{Bernoulli}\left(p_{i}\right),\\ p_{i}=F_{\lambda }\left(\eta_{i}\right),\\ \eta_{i}=x_{i}^{\prime }\beta ,\\ (\beta ,\lambda )∼\pi (\beta ,\lambda ), \end{array}$$

where *F*_λ_ represents the distributions previously introduced, ***β*** is the vector of regression coefficients, λ is the asymmetry parameter introduced by the power and reverse power transformations, and *π* is the prior distribution for the parameters ***β*** and λ.

In this work, it will be assumed that all parameters are independent, meaning that the prior distribution is given by *π*(***a***, ***b***) = *π*(***a***)*π*(***b***). Therefore, the priors used in the models will be based on the study by [4]. Additionally, following the authors’ recommendation, the parameter λ will be reparameterized as *δ* = log(λ), since this reparameterization enhances the efficiency of parameter estimates. The set of equations below describes the model used:

$$\begin{array}{c} Y_{i}|\beta ,\delta \overset{\text{ind.}}{∼}\text{Bernoulli}\left(p_{i}\right),\\ p_{i}=F_{\delta }\left(\eta_{i}\right),\\ \eta_{i}=x_{i}^{\prime }\beta ,\\ \beta_{j}\overset{\text{ind.}}{∼}\text{Normal}\left(0,10^{2}\right),\;j=1,2,\ldots ,k,\\ \delta ∼\text{Uniform}(-2,2), \end{array}$$

where *F*_*δ*_ represents the cdf of the reparameterized PDLomax or RPDLomax distribution.

Note that the prior distribution of *δ* is a uniform distribution on (−2, 2), that is, λ is restricted to the interval (*e*^−2^, *e*^2^) = (0.14, 7.39). The reason for this choice is that values outside this range have a very low probability of occurrence [16]. Moreover, the asymmetry of the power distributions remains practically constant when λ > 6 [13]. It can be observed that the asymmetry of the PDLomax and RPDLomax distributions is constant outside the interval established in this prior distribution. Despite this parameterization, the results in the following sections will be presented in terms of λ to maintain a direct connection with symmetric link functions and the success rate.

The posterior distribution for the binary regression models that have the asymmetry parameter λ is given by

$$\begin{array}{c} \pi (\beta ,\delta |X,y)\propto L(\beta ,\delta |X,y)\pi (\beta )\pi (\delta ), \end{array}$$

where *π*(***β***) is the prior distribution of ***β***, with $\beta_{j}\overset{\text{ind.}}{∼}\text{Normal}\left(0,10^{2}\right)$, for *j* = 1, 2, …, *k*; *π*(*δ*) is the prior distribution of *δ*, with *δ* = log(λ) ∼ Uniform(−2, 2); and L(β,δ|X,y) is the likelihood function of the parameters given the dataset, represented by the formula:

$$\begin{array}{c} L\left(\beta ,\delta |X,y\right)=\prod_{i=1}^{n}{\left[F_{\delta }\left(\eta_{i}\right)\right]}^{y_{i}}{\left[1-F_{\delta }\left(\eta_{i}\right)\right]}^{1-y_{i}}. \end{array}$$

Thus, combining the expressions described previously, the posterior distribution can be written as:

$$\begin{array}{cc} \pi (\beta ,\delta |X,y) & \propto \prod_{i=1}^{n}{\left[F_{\delta }\left(\eta_{i}\right)\right]}^{y_{i}}{\left[1-F_{\delta }\left(\eta_{i}\right)\right]}^{1-y_{i}}\prod_{j=1}^{p}\frac{1}{10\sqrt{2\pi }}\text{exp}\{-\frac{\beta_{j}^{2}}{2(10^{2})}\}\frac{1}{4}\\ & \propto \prod_{i=1}^{n}{\left[F_{\delta }\left(\eta_{i}\right)\right]}^{y_{i}}{\left[1-F_{\delta }\left(\eta_{i}\right)\right]}^{1-y_{i}}\prod_{j=1}^{p}\text{exp}\{-\frac{\beta_{j}^{2}}{2(10^{2})}\}. \end{array}$$ (1)

Note that the posterior distribution (1) is not similar to known distributions. Therefore, parameter estimation does not have an analytical solution and needs to be calculated computationally.

The Bayesian classifier approach is considered advantageous due to its use of the predictive posterior, which provides a probability distribution over the sensitivity probability of the *i*-th outcome, $P\left(Y_{i}=1|X_{i}\right)=p_{i}$. This allows for a more nuanced understanding of uncertainty in predictions, making it particularly useful when dealing with limited data (due to the possibility of incorporating priors). In contrast, the frequentist approach typically provides point estimates without capturing the full range of uncertainty (just a point estimation decision criteria), which can lead to less robust predictions. Therefore, the Bayesian approach, focusing on the predictive posterior, probabilistically offers a more comprehensive and complete uncertainty framework for making predictions.

All parameter estimation was performed using the stan package in the R software [20]. This program conducts its estimations using the No-U-Turn Sampler (NUTS) technique proposed by [21]. NUTS is a Markov chain Monte Carlo (MCMC) algorithm employed for Bayesian inference. It is a self-tuning variant of the Hamiltonian Monte Carlo (HMC) method, which efficiently explores the parameter space by utilizing gradient information to avoid random walks and provide faster convergence. NUTS uses a recursive algorithm to build a set of likely candidate points, covering a wide range of the target distribution, and it automatically stops when it starts returning to the same place. Empirically, NUTS performs at least as efficiently as HMC, and sometimes more efficiently, even when well specified, with the advantage that NUTS operates without user intervention [21]. A more detailed review of the NUTS algorithm applied to power and reverse power models can be found in the work of [15].

To compare the model fits, Bayesian metrics based on the posterior mean of deviance and the deviance from the posterior mean were used, represented by the following formulas:

$$\begin{array}{c} \bar{D}=\frac{1}{S}\sum_{s=1}^{S}D\left(\beta^{\left(s\right)},\delta^{\left(s\right)}\right),\;\;\text{with}\;\;D\left(\beta^{\left(s\right)},\delta^{\left(s\right)}\right)=-2\text{log}(p(y|\beta^{\left(s\right)},\delta^{\left(s\right)}), \end{array}$$

and

$$\begin{array}{c} \hat{D}=D(\frac{1}{S}\sum_{s=1}^{S}\beta^{\left(s\right)},\frac{1}{S}\sum_{s=1}^{S}\delta^{\left(s\right)}), \end{array}$$

for *s* = 1, …, *S*, where *S* is the size of the posterior sample. From these values, the effective number of parameters, $\rho_{d}=\bar{D}-\hat{D}$, can be calculated. Subsequently, measures such as deviance information criterion ($\text{DIC}=\bar{D}+\rho_{d}=2\bar{D}-\hat{D}$), expected Akaike information criterion ($\text{EAIC}=\bar{D}+2k$, where *k* is the number of the model’s parameters), and expected Bayesian information criterion ($\text{EBIC}=\bar{D}+k\text{log}\left(n\right)$, where *n* is the sample size, that is, the number of observations of the response variable *Y*) are computed. Additionally, Watanabe-Akaike information criterion ($\text{WAIC}=-2(\hat{\text{LPPD}}-{\hat{p}}_{\text{WAIC}})$, with $\hat{\text{LPPD}}=\sum_{i=1}^{n}\text{log}(S^{-1}\sum_{s=1}^{S}p\left(y_{i}|\beta^{\left(s\right)},\delta^{\left(s\right)}\right))$ and ${\hat{p}}_{\text{WAIC}}=2\sum_{i=1}^{n}(\text{log}(S^{-1}\sum_{s=1}^{S}p\left(y_{i}|\beta^{\left(s\right)},\delta^{\left(s\right)}\right))-S^{-1}\sum_{s=1}^{S}\text{log}(p(y_{i}|\beta^{\left(s\right)},\delta^{\left(s\right)})))$) and leave-one-out (LOO) metrics are considered.

The LOO metric, similar to WAIC, is a fully Bayesian metric. However, it has a high computational cost when dealing with very large samples. Therefore, [22] proposed the Pareto smoothed importance sampling leave-one-out (PSIS-LOO) cross-validation method. This metric can then be calculated as follows:

$$\begin{array}{c} {\hat{\text{ELPD}}}_{\text{PSIS-LOO}}=\sum_{i=1}^{n}\text{log}(\sum_{s=1}^{S}\frac{w_{i}^{\left(s\right)}\;p\left(y_{i}|\beta^{\left(s\right)},\delta^{\left(s\right)}\right)}{\sum_{s=1}^{S}w_{i}^{\left(s\right)}}), \end{array}$$

where

$$\begin{array}{c} w_{i}^{\left(s\right)}=\text{min}\;(r_{i}^{\left(s\right)},\frac{\sqrt{S}}{S}\sum_{s=1}^{S}r_{i}^{\left(s\right)}), \end{array}$$

with

$$\begin{array}{c} r_{i}^{\left(s\right)}=\frac{1}{p(y_{i}|\beta^{\left(s\right)},\delta^{\left(s\right)})}\propto \frac{p(\beta^{\left(s\right)},\delta^{\left(s\right)}|y_{-i})}{p(\beta^{\left(s\right)},\delta^{\left(s\right)}|y)}, \end{array}$$

for *s* = 1, …, *S*.

The smaller the value of all these metrics, the better the model fit.

To assess the model’s adequacy, quantile residuals, as proposed by [23], were utilized. When the model assumptions are met, these residuals follow a normal distribution, regardless of the response variable’s distribution.

## 5 Simulation studies

In this section, we will present simulation studies that aim to verify the accuracy of the Bayesian estimation procedure (Section 5.1), and evaluate whether the proposed models can perform better than the logistic regression model in different imbalanced scenarios (Section 5.2).

### 5.1 Parameter recovery

In order to assess the ability of the proposed models to estimate their parameters, a simulation study was conducted. In this study, 100 random samples of size *n* = {500, 1, 000, 2, 000} were generated for each of the proposed models and also for the logistic model (used for comparison). For models that include the asymmetry parameter (λ), four additional scenarios were considered: λ = {0.25, 0.5, 2, 4}. The covariate *X* was simulated from a uniform distribution on the interval (−3, 3), and the regression coefficients were fixed at ***β*** = (*β*_0_, *β*_1_) = (0, 1).

To estimate the parameters using the stan software, 200 iterations, 4 chains, and 100 samples of warm-up were considered for each chain. Consequently, for each replica, 400 samples of each estimated parameter were generated. The metrics used to assess the models’ performance were bias and root mean squared error (RMSE), which are defined, respectively, as follows:

$$\begin{array}{c} \text{Bias}\left(\hat{\theta }\right)=\frac{1}{R}\sum_{r=1}^{R}\left({\hat{\theta }}^{\left(r\right)}-\theta \right)\;\;\text{and}\;\;\text{RMSE}\left(\hat{\theta }\right)=\sqrt{\frac{1}{R}\sum_{r=1}^{R}{\left({\hat{\theta }}^{\left(r\right)}-\theta \right)}^{2}}, \end{array}$$

where *θ* ∈ ***θ*** = (λ, ***β***) (is one of the three parameters), *R* is the number of replications in the simulation (in this case, *R* = 100), *θ* represents the true parameter value, and ${\hat{\theta }}^{\left(r\right)}$ stands for the posterior mean of parameter *θ* in replication *r*.

Furthermore, the quality of the interval estimation was verified, observing the relative frequency of times in which the true parameter (*θ*) was contained in percentiles 2.5% and 97.5%, that is, the proportion of times in which the original parameter was between percentiles 2.5% and 97.5% of the samples of the posterior distribution, here called coverage probability (CP). That is,

$$\begin{array}{cc} & \text{CP}=\frac{1}{R}\sum_{r=1}^{R}I\left(\theta \in \left[\text{LL},\text{UL}\right]\right), \end{array}$$

where LL and UL are, respectively, the lower (2.5%) and upper (97.5%) limits of the posterior samples for each replicate. In other words, we calculated the coverage of the 95% credibility interval of MCMC chains. Hence, it is expected that, in 95% of cases, the real value of the parameter will be contained within this range.

Table 1 reveals that in most models, the bias of the estimator ${\hat{\beta }}_{0}$ decreases as the sample size increases. The models dealing with more imbalanced data, i.e., with λ = 0.25 and λ = 4, show higher bias and higher RMSE. The same holds for *β*_1_: its bias also decreases as the sample size increases, as does its RMSE. In comparison to the logistic model, in smaller sample sizes, the proposed models exhibit more bias in parameter recovery. However, as the sample size grows, the difference between these models becomes negligible.

**Table 1:** Bias, RMSE and CP calculated for the parameters *β*_0_, *β*_1_, and λ.

Model	*n*	*β*_0_			*β*_1_			λ		
		Bias	RMSE	CP	Bias	RMSE	CP	Bias	RMSE	CP
Logistic	500	0.009	0.011	0.96	0.016	0.009	0.95	-	-	-
	1,000	0.007	0.003	0.91	0.007	0.004	0.95	-	-	-
	2,000	0.002	0.008	0.91	0.002	0.001	0.97	-	-	-
DLomax	500	0.011	0.016	0.96	0.066	0.037	0.96	-	-	-
	1,000	0.007	0.009	0.96	0.049	0.020	0.92	-	-	-
	2,000	0.001	0.004	0.95	0.020	0.007	0.93	-	-	-
PDLomax(λ = 0.25)	500	0.964	9.806	0.95	0.785	1.059	0.88	0.044	0.345	0.93
	1,000	0.558	3.448	0.94	0.410	0.395	0.90	0.023	0.026	0.91
	2,000	0.317	1.862	0.95	0.176	0.128	0.94	0.009	0.011	0.96
PDLomax(λ = 0.5)	500	-0.186	0.279	0.93	0.307	0.242	0.90	-0.039	0.009	0.91
	1,000	-0.033	0.140	0.93	0.161	0.081	0.87	-0.012	0.007	0.91
	2,000	-0.028	0.064	0.95	0.062	0.020	0.94	-0.005	0.004	0.91
PDLomax(λ = 2)	500	-0.030	0.119	0.92	0.096	0.035	0.96	-0.002	0.166	0.95
	1,000	-0.014	0.027	0.95	0.053	0.016	0.97	0.006	0.040	0.94
	2,000	0.009	0.014	0.93	0.022	0.006	0.96	0.009	0.019	0.95
PDLomax(λ = 4)	500	0.103	0.101	0.97	0.382	0.258	0.90	0.746	1.496	0.96
	1,000	0.200	0.110	0.89	0.224	0.105	0.86	0.748	1.545	0.83
	2,000	0.143	0.066	0.86	0.130	0.053	0.87	0.513	0.898	0.87
RPDLomax(λ = 0.25)	500	-0.733	7.506	0.95	0.640	0.801	0.89	0.026	0.223	0.95
	1,000	-0.385	2.266	0.95	0.306	0.269	0.91	0.015	0.019	0.93
	2,000	-0.204	0.862	0.93	0.132	0.095	0.96	0.011	0.005	0.96
RPDLomax(λ = 0.5)	500	0.112	0.494	0.87	0.297	0.258	0.92	-0.008	0.026	0.89
	1,000	0.028	0.112	0.96	0.118	0.061	0.93	-0.007	0.005	0.94
	2,000	0.032	0.037	0.97	0.046	0.019	0.94	-0.008	0.002	0.95
RPDLomax(λ = 2)	500	-0.047	0.102	0.91	0.088	0.032	0.96	0.010	0.167	0.92
	1,000	-0.027	0.042	0.92	0.063	0.024	0.89	0.052	0.094	0.93
	2,000	0.003	0.011	0.99	0.019	0.008	0.96	-0.003	0.016	0.96
RPDLomax(λ = 4)	500	-0.182	0.124	0.93	0.338	0.199	0.87	0.906	1.848	0.98
	1,000	-0.187	0.093	0.91	0.225	0.115	0.84	0.740	1.438	0.85
	2,000	-0.135	0.076	0.89	0.140	0.065	0.88	0.454	1.028	0.86

Regarding the parameter λ, a slight increase in bias and RMSE is noticeable from *n* = 500 to *n* = 1, 000 when λ = 4. This might occur because, as stated by [13], the relationship between an increase in λ and asymmetry is not linear. Beyond a certain point, any increase in λ results in insignificant increments in asymmetry. Nevertheless, a strong downward trend in both bias and RMSE is evident when *n* = 2, 000, indicating the potential for reducing bias in the model asymptotically. For the remaining models with the parameter λ, both bias and RMSE decrease as the sample size increases.

Finally, the credibility intervals for the parameters of all models seem to have a reasonable behavior, given the number of repetitions of the experiment; however, it is notable that the CP is smaller when λ = 4.

### 5.2 Misspecification

As in the work of [13], imbalanced data were generated based on the power Cauchy model, with fixed regression coefficients *β*_0_ and *β*_1_ set to ***β*** = (*β*_0_, *β*_1_) = (0, 1). The (sole) covariate *X* was simulated from a uniform distribution on the interval (−3, 3). Four different scenarios were simulated with varying levels of imbalance, considering the parameter λ = {0.25, 0.5, 2, 4} of the power Cauchy distribution. The binary regression model using the power Cauchy link function is presented below:

$$\begin{array}{c} Y_{i}|X_{i}=x_{i}\overset{\text{ind.}}{∼}\text{Bernoulli}\left(p_{i}\right),\\ p_{i}={\left(\frac{1}{\pi }\text{arctan}\left(\beta_{0}+\beta_{1}x_{i}\right)+\frac{1}{2}\right)}^{\lambda }. \end{array}$$

In this experiment, 100 samples with the power Cauchy distribution were generated following the structure outlined above, each containing 5,000 observations. Table 2 displays the degree of imbalance in each sample.

**Table 2:** Mean proportion of 1’s, for λ = {0.25, 0.5, 2, 4}, of the samples of the power Cauchy model.

	λ = 0.25	λ = 0.5	λ = 2	λ = 4
Mean proportion of 1’s	0.800	0.661	0.329	0.198

To compare the fit of the proposed models with the logistic regression model, the WAIC and LOO metrics were examined. These metrics were chosen because they tend to perform better in model selection than other metrics such as DIC, as these only consider point estimates, while the WAIC and LOO metrics take into account the entire posterior distribution of the parameters [24]. In addition, they are fully Bayesian measures. From these metrics, the means of LOO and WAIC in each scenario ($\bar{\text{LOO}}$ and $\bar{\text{WAIC}}$, respectively), the percentage of times that the metric of each link is less than the logistic link (%_LOO_ and %_WAIC_), and the variance of each of these metrics ($s_{\text{LOO}}^{2}$ and $s_{\text{WAIC}}^{2}$) were calculated. That is,

$$\begin{array}{ll} \bar{\text{LOO}}=\frac{1}{R}\overset{R}{\underset{r=1}{\sum }}\;{\text{LOO}}^{\left(r\right)}, & \bar{\text{WAIC}}=\frac{1}{R}\overset{R}{\underset{r=1}{\sum }}\;{\text{WAIC}}^{\left(r\right)},\\ {\%}_{\text{LOO}}=\frac{1}{R}\overset{R}{\underset{r=1}{\sum }}\;I({\text{LOO}}^{\left(r\right)}<{\text{LOO}}_{\text{log}.}^{\left(r\right)}), & {\%}_{\text{WAIC}}=\frac{1}{R}\overset{R}{\underset{r=1}{\sum }}\;I({\text{WAIC}}^{\left(r\right)}<{\text{WAIC}}_{\text{log}.}^{\left(r\right)}),\\ s_{\text{LOO}}^{2}=\frac{1}{R-1}\overset{R}{\underset{r=1}{\sum }}{\left({\text{LOO}}^{\left(r\right)}-\bar{\text{LOO}}\right)}^{2}, & s_{\text{WAIC}}^{2}=\frac{1}{R-1}\overset{R}{\underset{r=1}{\sum }}{\left({\text{WAIC}}^{\left(r\right)}-\bar{\text{WAIC}}\right)}^{2}, \end{array}$$

where *R* is the number of simulation replicas (in this case, *R* = 100), and I denotes the indicator function.

Table 3 shows that in all cases, at least one of the proposed models (PDLomax and RPDLomax) performed better than the logistic regression. When λ = 0.25, the RPDLomax model, even with LOO and WAIC values higher than logistic regression, still outperforms it in 58% of cases in both metrics. When λ = 0.5, it can be observed that the PDLomax and RPDLomax models have lower WAIC and LOO values than the logistic model, performing better in over 60% of cases, with the PDLomax model outperforming all (66% of success compared to logistic regression). When λ = 2, the PDLomax and RPDLomax models present lower average LOO and WAIC values than logistic regression and also outperform it most of the time; in this case, the PDLomax model had better results (LOO and WAIC were lower than the logistic model in 60% of cases). Finally, at λ = 4, the PDLomax model, even with WAIC and LOO values higher than the logistic model, has lower LOO in 65% of cases and lower WAIC in 62% of cases.

**Table 3:** Comparative Bayesian measures of the proposed models and binary logistic regression.

Link	$\bar{\text{LOO}}$	$s_{\text{LOO}}^{2}$	%_LOO_	$\bar{\text{WAIC}}$	$s_{\text{WAIC}}^{2}$	%_WAIC_
λ = 0.25						
Logit	5,007.371	71.147	-	5,007.343	71.147	-
DLomax	5,007.349	71.169	49	5,007.320	71.169	49
PDLomax	5,008.029	70.471	44	5,008.002	70.471	45
RPDLomax	5,008.471	70.813	58	5,008.448	70.811	58
λ = 0.5						
Logit	6,403.237	39.623	-	6,403.208	39.622	-
DLomax	6,403.268	39.645	40	6,403.239	39.645	41
PDLomax	6,403.053	39.570	66	6,403.025	39.571	66
RPDLomax	6,403.113	39.602	62	6,403.086	39.602	62
λ = 2						
Logit	6,334.080	38.417	-	6,334.051	38.417	-
DLomax	6,333.970	38.384	60	6,333.942	38.384	60
PDLomax	6,333.958	38.358	60	6,333.931	38.359	60
RPDLomax	6,333.960	38.404	59	6,333.932	38.404	59
λ = 4						
Logit	4,977.608	65.323	-	4,977.579	65.322	-
DLomax	4,977.568	65.304	51	4,977.540	65.305	52
PDLomax	4,979.453	65.368	65	4,979.433	65.373	62
RPDLomax	4,978.640	65.189	42	4,978.616	65.189	43

## 6 Applications

This section presents two applications that were developed in order to illustrate the performance of the DLomax, PDLomax, and RPDLomax link functions on real data. First, the proposed models were applied to a database with images of diseased trees (Section 6.1), and then the effect of these new link functions was studied on a blood donation database (Section 6.2).

### 6.1 Wilt dataset

This study considers a dataset comprised of image segments resulting from the pansharpening technique, as described in the work of [25], for the detection of diseased pine and oak trees. This dataset was created because in Japan, beetles that feed on pine and oak trees are responsible for the majority of damage to forested areas, as they transmit diseases to the trees, causing them to wither. Hence, rapid detection, removal, or treatment of newly infected trees is necessary to prevent the beetles from emerging the following year and spreading their diseases. The discoloration of the foliage is a clear sign of infection, so the detection of a diseased tree is usually associated with the detection of a discolored tree. Due to the fact that the number of diseased trees was much smaller compared to the number of healthy trees in the study area, collecting images of diseased trees was more challenging and time-consuming. Consequently, an imbalanced dataset was constructed.

This dataset was introduced in the paper [25] and is available in the UCI repository [26]. For this study, the validation dataset, which consists of 500 observations, was considered. Below is a brief description of the variables in the dataset (given the complexity of the subject, more information about the variables can be found in [25]):

- *GLCM_Pan*: gray-level co-occurrence matrix (GLCM) mean texture;
- *Mean_G*: mean green value;
- *Mean_R*: mean red value;
- *Mean_NIR*: mean near-infrared (NIR) value;
- *SD_Pan*: standard deviation of the panchromatic (Pan) band;
- *Y*: binary variable indicating whether the tree is diseased (1) or not (0).

Table 4 presents the descriptive statistics for each of the variables. This dataset exhibits a 37.4% success (observed diseased trees, *Y* = 1) rate.

**Table 4:** Descriptive measures of the Wilt dataset. SD = standard deviation.

Variable	Mean	Min.	Max.	SD
*GLCM_Pan*	127.07	81.12	167.94	10.67
*Mean_G*	209.80	117.20	1848.90	78.68
*Mean_R*	107.74	50.58	1594.58	71.77
*Mean_NIR*	453.70	144.90	1597.30	156.20
*SD_Pan*	20.64	5.77	62.39	6.76

When analyzing the data, it was found that the variables *Mean_G* and *Mean_R* had a correlation of 0.98. We then removed the variable *Mean_G* from the subsequent analysis, since it was more correlated with the other variables. Therefore, the models were adjusted using the standardized variables *GLCM_Pan*, *Mean_R*, *Mean_NIR* and *SD_Pan*. Once again, the models were fitted using the stan package of the R software. In each case, 5,000 iterations, 4 chains, and 2,500 warm-up iterations were considered. In almost all cases (except for the PDLomax distribution), convergence was achieved based on the potential scale reduction statistic ($\hat{R}$) of [27]. The PDLomax model encountered convergence issues, potentially influenced by several factors, particularly the choice of priors for λ and ***β***.

It is evident from Table 5 that the RPDLomax model stands out when compared to the other models (including the well-known logistic, probit, cauchit, loglog, and complementary log-log or cloglog models). This model had significantly lower values for DIC, EAIC, EBIC, LOO, and WAIC, demonstrating its superior suitability for the dataset compared to the other models presented. The DLomax model, on the other hand, was the second-best performing model, obtaining the second lowest measures for DIC, EAIC, EBIC, LOO, and WAIC. The cauchit model, although achieving metrics similar to the DLomax model, did not exhibit any measures superior to the RPDLomax and DLomax models.

**Table 5:** Comparison metrics of the models fitted to the Wilt dataset.

Model	*ρ*_*d*_	$\bar{D}$	$\hat{D}$	DIC	EAIC	EBIC	LOO	WAIC
DLomax	4.658	532.591	527.933	537.249	542.591	563.664	537.874	537.863
PDLomax	-	-	-	-	-	-	-	-
RPDLomax	3.985	428.726	424.741	432.711	440.726	466.013	441.044	441.031
Logistic	5.059	657.361	652.302	662.420	667.361	688.434	688.290	700.992
Probit	5.003	660.494	655.492	665.497	670.494	691.567	675.818	672.110
Cauchit	4.916	543.991	539.076	548.907	553.991	575.064	549.015	549.008
loglog	5.060	635.796	630.735	640.856	645.796	666.869	656.906	667.428
cloglog	4.919	662.544	657.625	667.463	672.544	693.617	670.433	668.231

As the RPDLomax model was chosen, Table 6 provides descriptive statistics of the posterior distribution samples for this model. In this table, it can be observed that the 90% credibility interval for the skewness parameter λ does not encompass the one value, suggesting that the current model cannot be reduced to the base model (DLomax). Additionally, the 90% credibility intervals for the *β*_1_ and *β*_4_ parameters encompass the zero value; thus, the *GLCM_Pan* and *SD_Pan* covariates are not significant. Despite that, these covariates were kept in the model as they are important for ensuring that the model’s residuals meet the assumption of normality.

**Table 6:** Descriptive measures of the parameters of the RPDLomax model fitted to the Wilt dataset.

Covariate	Parameter	Mean	SD	Median	Percentile 5%	Percentile 95%
Intercept	*β*_0_	26.110	11.423	24.445	10.738	47.329
*GLCM_Pan*	*β*_1_	1.142	1.732	1.026	-1.464	4.141
*Mean_R*	*β*_2_	137.094	53.400	128.895	64.941	236.957
*Mean_NIR*	*β*_3_	-19.340	7.268	-18.330	-32.818	-9.549
*SD_Pan*	*β*_4_	0.206	2.120	0.332	-3.300	3.307
Skewness	λ	0.256	0.038	0.251	0.203	0.327

Using the posterior mean as the point estimate for the parameters, the adopted model can be represented by the formula:

$$\begin{array}{c} {\hat{\eta }}_{i}=26.110+1.142\;X_{i1}+137.094\;X_{i2}-19.340\;X_{i3}+0.206\;X_{i4},\\ {\hat{p}}_{i}=\{\begin{array}{cc} 1-{\left[1-\frac{1}{2(1-{\hat{\eta }}_{i})}\right]}^{0.256}, & {\hat{\eta }}_{i}\leq 0,\\ 1-{\left[\frac{1}{2(1+{\hat{\eta }}_{i})}\right]}^{0.256}, & {\hat{\eta }}_{i}>0, \end{array}\\ {\hat{Y}}_{i}|X_{i}\overset{\text{ind.}}{∼}\text{Bernoulli}\left({\hat{p}}_{i}\right), \end{array}$$

where *X*_1_, *X*_2_, *X*_3_, and *X*_4_ are, respectively, the standardized *GLCM_Pan*, *Mean_R*, *Mean_NIR*, and *SD_Pan* variables.

**Fig 3:**
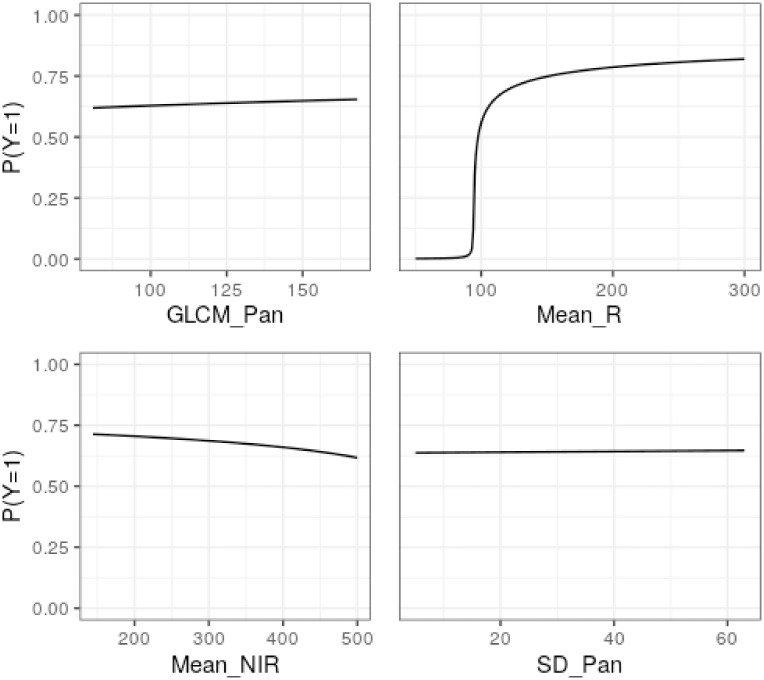
Nonlinear effect of each variable on the probability that a tree is diseased (P(Y=1)), on average, when the other variables are constant, based on the adjusted RPDLomax model (Wilt dataset).

Observing the signs of the parameters, it can be interpreted that:

- As the variables *GLCM_Pan*, *Mean_R* and *SD_Pan* increase, the probability of the tree being diseased also increases;
- As the variable *Mean_NIR* increases, the probability of the tree being diseased decreases;
- The variable *Mean_R* plays a significant role in calculating the probability of the tree being diseased, given the magnitude of its associated parameter. This makes sense because dry trees lose their green color and exhibit more reddish tones;

In addition to interpreting the signs of the parameters, the impact can be observed of the variation of each variable on the probability of a tree being diseased. In Fig 3, the effect of the variation of each variable is presented, maintaining the others at their average value.

From Fig 3, the significant impact of the variable *Mean_R* can be seen, in which there is a sharp increase in the probability of success (diseased tree) in the range between 90 and 100. When *Mean_R* equals 90, the probability of success is 0.015, while for *Mean_R* equals 100, the probability of success is 0.558. On the other hand, the other variables seem to have a smaller influence on the probability of success as their curves show little variation.

**Fig 4:**
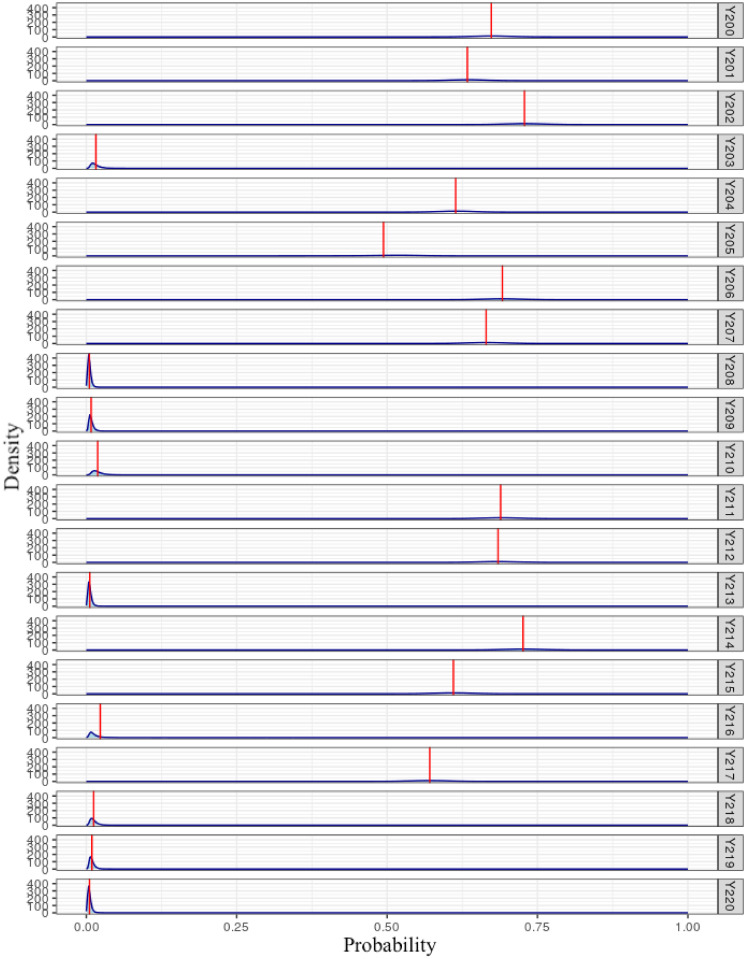
Density of the predictive probabilities estimated by the RPDLomax model adopted in the first application (Wilt dataset), for observations #200 to 220. The red line represents the average of the estimated diseased tree probabilities, $\hat{P}\left(Y_{i}=1|X_{i}\right)={\hat{p}}_{i}$.

The interpretations provided above, Bayesian Statistics also allows us to interpret the probabilities of success (*Y* = 1) of each of the observations. In Fig 4, it is noted that some observations have a very low probability of success, and their distributions are concentrated in an interval close to 0, while other observations, in turn, are concentrated in points closer to the center, or have a more flattened distribution. Thus, observations can be identified that have a greater degree of uncertainty in their classification.

**Fig 5:**
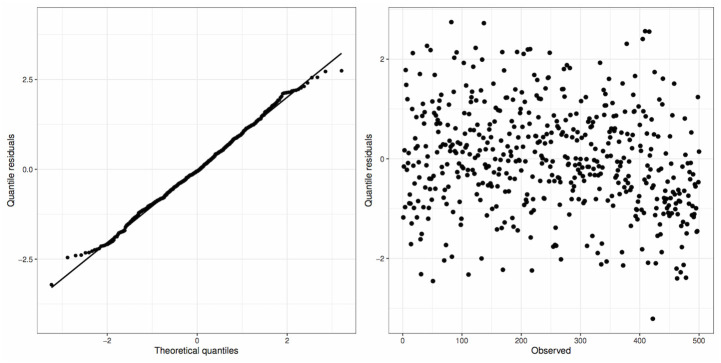
Plots of the quantile residuals of the RPDLomax model fitted to the Wilt dataset.

The residuals of this model are displayed graphically in Fig 5. It can be observed that the residuals behave as expected, showing no signs that they do not follow a normal distribution (left panel). Additionally, it can be noted that the residuals appear to be distributed randomly, with no strong evidence of a trend or changes in variance (right panel).

**Fig 6:**
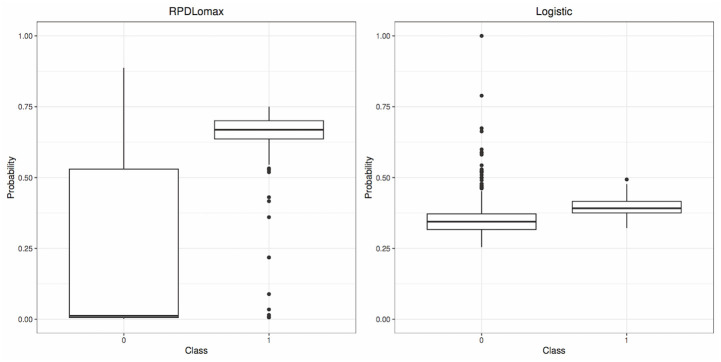
Boxplots of the estimated mean predictive probabilities for each observation, based on the RPDLomax model and the Logistic model for each class (Wilt dataset).

In Fig 6, it is clear that the RPDLomax model assigns low mean posterior predictive probabilities to failure and higher mean posterior predictive probabilities to success (left panel). This is in contrast to the logistic regression model, which does not clearly distinguish between the mean predictive probabilities for the two classes (right panel). In Table 7, the median posterior predictive probability for the not diseased tree group, $P(\hat{Y}=1|Y=0,X)$, based on the RPDLomax model was 1.2%, while for the Logistic model was 34.4%. In comparison, the median predictive probability for the diseased tree, $P(\hat{Y}=1|Y=1,X)$, based on the RPDLomax model was 66.9%, versus 39.2% for the Logistic model.

**Table 7:** Descriptive statistics (minimum, first quartile, median, mean, third quartile, maximum) of probabilities for the RPDLomax and Logistic models by class (Wilt dataset).

Model	Class	Min.	1st Qu.	Median	Mean	3rd Qu.	Max.
RPDLomax	Success	0.006	0.636	0.669	0.637	0.701	0.750
	Failure	0.002	0.006	0.012	0.214	0.530	0.887
Logistic	Success	0.322	0.375	0.392	0.395	0.416	0.493
	Failure	0.255	0.317	0.344	0.360	0.372	1.000

### 6.2 Blood donation dataset

The database used was introduced and analyzed by [28], and is available in the UCI repository [26]. This dataset comprises 748 random samples of blood donor data from the Blood Transfusion Center in Hsin-Chu City, Taiwan, with the following variables:

- *Recency*: number of months since the last donation;
- *Frequency*: total number of donations made by the donor;
- *Time*: time, in months, since the first donation;
- *Monetary*: total, in milliliters (ml), of blood donated since the first donation;
- *Y*: binary variable indicating whether he/she donated blood (1—yes, 0—no) in March 2007.

Table 8 shows the descriptive statistics for each of these variables. This dataset exhibits a 23% success (1’s) rate.

**Table 8:** Descriptive measures of the Blood Donation dataset.

Variable	Mean	Min.	Max.	SD
*Recency*	9.5	0	74	8.1
*Frequency*	5.5	1	50	5.8
*Time*	34.3	2	98	24.4
*Monetary*	1,378.7	250	12,500	1,459.8

In the initial analysis, it was observed that the variables *Frequency* and *Monetary* had a correlation of 1 (perfect positive correlation). This occurs because 250 ml of blood is donated with each donation, therefore the *Monetary* variable, which represents the total donated blood, is nothing more than the *Frequency* variable multiplied by 250. Therefore, the decision was made to exclude the *Monetary* variable from the model. In addition to the correlation between these two variables, no other correlations were found that would hinder the model fitting.

Thus, the models were adjusted considering the standardized covariates *Recency*, *Frequency*, and *Time* to predict the variable *Y*. The stan package in the R software was used for parameter estimation. For each distribution, 5,000 iterations, 4 chains, and 2,500 warm-up iterations were considered. Convergence was achieved in all distributions based on the potential scale reduction statistic ($\hat{R}$) by [27].

In Table 9, it can be observed that the RPDLomax model achieved the lowest values in the DIC, EAIC, LOO, and WAIC metrics, proving to be the model that performed better in most of the metrics. The cauchit model also showed satisfactory performance; however, it only outperformed the RPDLomax model in the EBIC metric. On the other hand, the other models proposed in this work, PDLomax and DLomax, although not performing as well as the RPDLomax model, demonstrated superiority over most traditional models, as they showed lower DIC, EAIC, LOO, and WAIC values than the logistic, probit, loglog, and cloglog models.

**Table 9:** Comparison metrics of the models fitted to the Blood Donation dataset.

Model	*ρ*_*d*_	$\bar{D}$	$\hat{D}$	DIC	EAIC	EBIC	LOO	WAIC
DLomax	4.179	708.800	704.620	712.979	716.800	735.269	712.960	712.953
PDLomax	3.545	707.867	704.322	711.412	717.867	740.954	713.474	713.474
RPDLomax	4.963	706.123	701.159	711.086	716.123	739.210	711.542	711.532
Logistic	3.976	711.861	707.885	715.837	719.861	738.330	716.279	716.271
Probit	3.962	713.551	709.589	717.513	721.551	740.020	718.261	718.201
Cauchit	4.111	708.511	704.400	712.622	716.511	734.981	712.855	712.852
loglog	3.995	715.224	711.229	719.219	723.224	741.694	719.814	719.793
cloglog	4.016	715.178	711.162	719.193	723.178	741.647	721.906	720.949

Considering the model comparison criteria and predictive evaluation, the RPDLomax model was chosen. Therefore, Table 10 presents the descriptive measures of the posterior samples of this model’s parameters. Note that all parameters (coefficients) *β*’s are significant, as none of the 90% credibility intervals for them include the zero value. Additionally, the 90% credibility interval for λ does not include the one value, indicating that this parameter is important for the fit, and the current model cannot be reduced to the base model (DLomax).

**Table 10:** Descriptive measures of the parameters of the RPDLomax fitted to the Blood Donation dataset.

Covariate	Parameter	Mean	SD	Median	Percentile 5%	Percentile 95%
Intercept	*β*_0_	-1.410	0.479	-1.352	-2.281	-0.747
*Recency*	*β*_1_	-1.381	0.356	-1.343	-2.021	-0.872
*Frequency*	*β*_2_	1.422	0.349	1.396	0.902	2.029
*Time*	*β*_3_	-0.889	0.246	-0.870	-1.319	-0.520
Skewness	λ	0.679	0.148	0.656	0.476	0.955

It was chosen to use the posterior mean as the point estimate for the parameters. Thus, the adopted model can be represented as follows:

$$\begin{array}{c} {\hat{\eta }}_{i}=-1.410-1.381\;X_{i1}+1.422\;X_{i2}-0.889\;X_{i3},\\ {\hat{p}}_{i}=\{\begin{array}{cc} 1-{\left[1-\frac{1}{2(1-{\hat{\eta }}_{i})}\right]}^{0.679}, & {\hat{\eta }}_{i}\leq 0,\\ 1-{\left[\frac{1}{2(1+{\hat{\eta }}_{i})}\right]}^{0.679}, & {\hat{\eta }}_{i}>0, \end{array}\\ {\hat{Y}}_{i}|X_{i}\overset{\text{ind.}}{∼}\text{Bernoulli}\left({\hat{p}}_{i}\right), \end{array}$$

where *X*_1_, *X*_2_, and *X*_3_ are, respectively, the standardized *Recency*, *Frequency*, and *Time* variables.

Observing the signs of the parameters, it can be interpreted that:

- As the number of months since the last donation (*Recency*) increases, the probability of the donor donating blood on the specified date decreases;
- When the number of donations made by the donor (*Frequency*) increases, the probability of he/she donating in the period also increases;
- As the time in months since the first donation (*Time*) increases, the probability of donation in March 2007 decreases.

These interpretations make sense, as donors who made their first donation a long time ago, donated infrequently, and have not donated blood for a long time; they represent a profile of sporadic donors. On the other hand, donors with a high number of donations represent the profile of regular donors.

**Fig 7:**
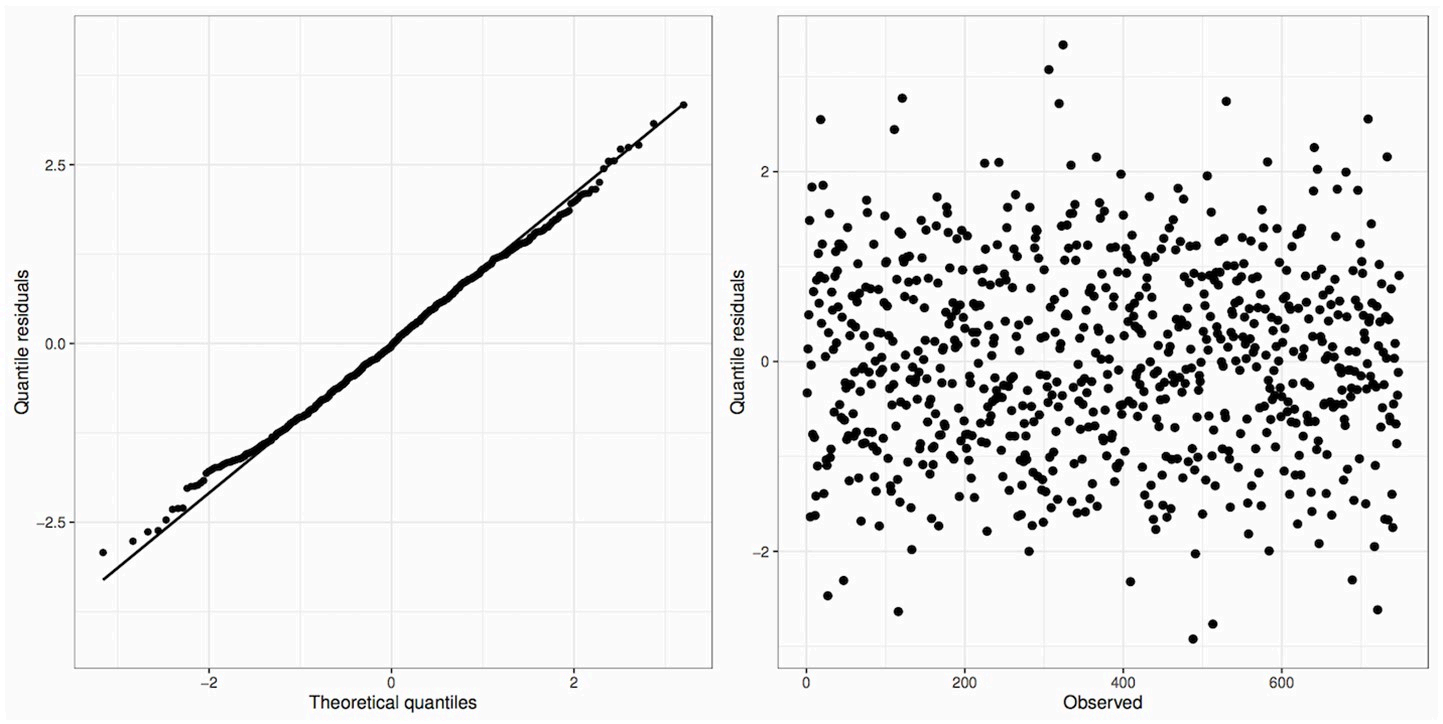
Plots of the quantile residuals of the RPDLomax model fitted to the Blood Donation dataset.

In Fig 7, the quantile residuals exhibit the expected behavior, showing no indications of deviating from a normal distribution (left panel). Furthermore, the residuals appear to be distributed randomly, lacking compelling evidence of any discernible trend or variations in variance (right panel).

**Fig 8:**
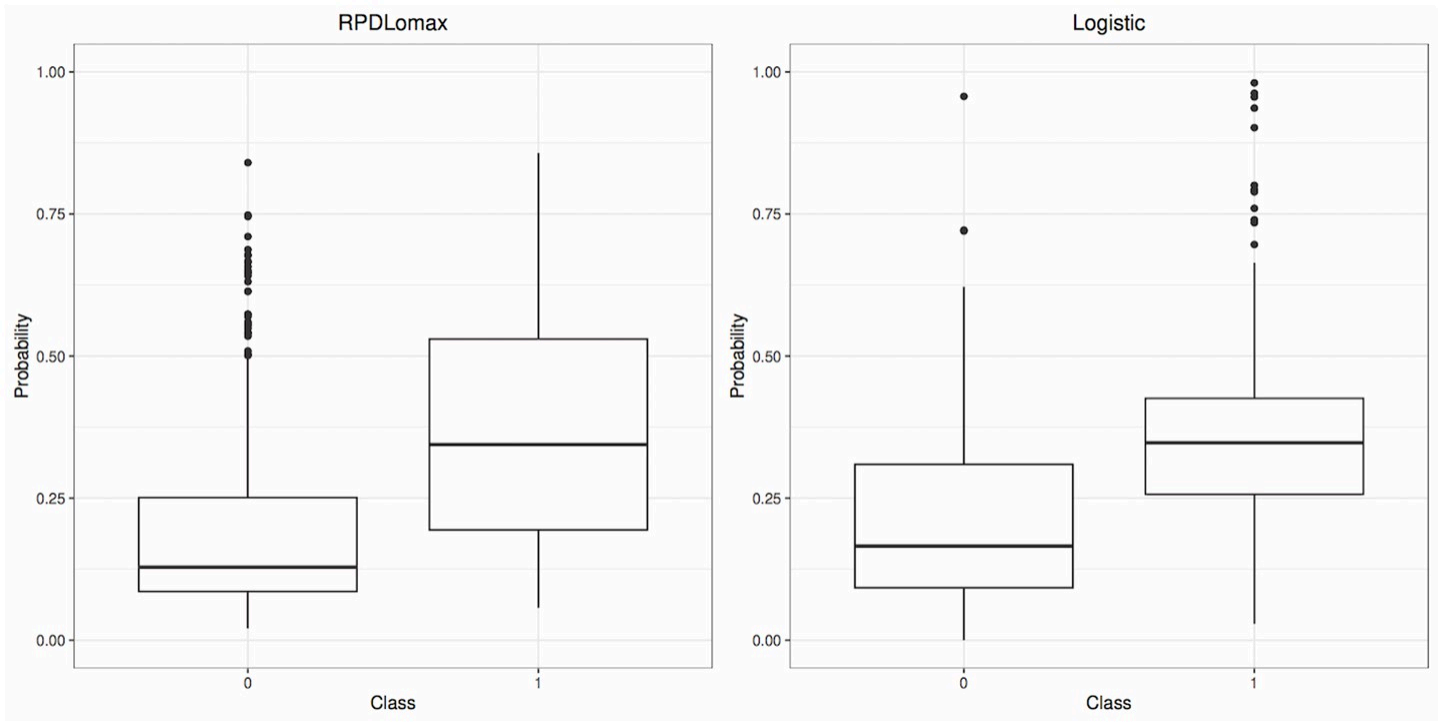
Boxplots of the estimated mean predictive probabilities for each observation, based on the RPDLomax model and the Logistic model for each class (Blood Donation dataset).

Turning our attention to Fig 8, one can discern a shift in the mean predictive distribution of success (1) versus the failure (0) mean predictive probabilities when transitioning from the logistic model (right panel) to the RPDLomax model (left panel). In the RPDLomax model, the medians of these predictives are more distinctly (separable), resulting in a heightened distinction between the probabilities associated with each class.

## 7 Discussion

In this study, the RPDLomax model was identified as the optimal choice due to its superior selection metrics (LOO, WAIC, and DIC) and satisfactory fit, with residuals conforming to necessary assumptions. This finding is consistent with literature indicating that models with asymmetric links often outperform those with symmetric links in real-world data applications [5, 7].

The reverse transformation exhibited outstanding performance in predicting real data, supported by multiple studies [2, 4, 14, 16]. In most cases, the reverse power transformation outperformed the power transformation, except in [10], where the latter achieved better results. Therefore, while both transformations yield similar outcomes, the reverse transformation generally offers a slight performance advantage.

Furthermore, the Bayesian approach for parameter fitting and estimation was explored. However, it is noted that many studies in this field do not fully utilize all the available information from this approach, such as the probability distribution of success for each sample, as illustrated in Fig 4.

### 7.1 Implication

The significance and novelty of this work lie in the introduction of new asymmetric classification functions that outperform traditional link functions such as logit, cauchit, probit, loglog, and cloglog. These novel functions offer a robust option for classifying binary imbalanced data and can be integrated into the R workflow. The simulation studies conducted in this paper demonstrated that these models can surpass logistic regression in various scenarios. Moreover, the simulations indicated that the parameters of these models can be recovered with low bias and reduced variance, particularly in larger samples.

The models were implemented using a Bayesian approach, which provides several advantages. Bayesian methods facilitate the incorporation of prior knowledge into the analysis through the use of prior distributions. They also offer a natural mechanism for quantifying uncertainty in parameter estimates and predictions. Additionally, Bayesian methods can exhibit greater robustness than frequentist methods when dealing with small sample sizes, owing to the ability to utilize informative prior distributions.

### 7.2 Limitations

Some limitations of this study should be considered. First, the number of replications and iterations in the simulations was limited. Increasing these in future work could improve the reliability of the results. The PDLomax model demonstrated promising performance with the second database but failed to converge in the first application. This suggests that the choice of priors impacts model performance. Future studies should investigate the use of less restrictive priors to improve convergence.

Moreover, the models were tested only on small datasets with a limited number of numerical features. The performance of these models on larger datasets with more features, including categorical variables, remains uncertain. Additionally, while the Bayesian approach offers a rich interpretative framework, it is computationally intensive, particularly for large-scale datasets. Future research should aim to optimize computational efficiency, or alternatively, consider a frequentist approach for handling big data.

### 7.3 Future work

In future studies, these presented models can be explored for new applications, especially in larger datasets. Additionally, the asymmetric link functions can be used as activation functions in neural networks, as [29] demonstrated that asymmetric activation functions can improve time series prediction with neural networks in their work. Furthermore, a performance comparison (also using, for instance, the evaluation metrics obtained from the confusion matrix, as well as an appropriate validation scheme) of the presented models with other asymmetric links (e.g., the ones based on other power and reverse power distributions) can be conducted. Moreover, implementing these models in R packages would enhance their accessibility and usability within the research community. Additionally, exploring alternative priors for λ could provide further refinement to the models.

## 8 Conclusion

In this work, new approaches to modeling imbalanced data were presented by introducing new link functions for binary regression. These novel link functions were created by applying the transformation proposed by [4] to the double Lomax distribution (DLomax) [17], in order to generate the power double Lomax (PDLomax) and reverse power double Lomax (RPDLomax) distributions. Despite their various applications, the Lomax distribution and its extensions had not been explored in the context of binary regression until now, making this work a novel and significant contribution to the literature. Additionally, the evidence pointed out the advantages of using the Bayesian classification approach by associating each event with a predictive posterior probability. Then, the Lomax Bayesian learning overcame the Logistic classification for differentiating binary data in imbalanced tasks.

Two simulation studies were carried out to assess the models’ ability to recover parameters and their fit quality under misspecification scenarios. The first study indicated that the proposed models and Bayesian estimation procedure are efficient at parameter recovery and exhibit reduced estimation bias as the sample size increases. In the second study, it was observed that the proposed models outperformed logistic regression in terms of model fit quality as evaluated by LOO and WAIC metrics, both in scenarios with moderate and severe asymmetry.

The proposed models were also applied to two imbalanced real datasets. The first database pertains to the classification of potential blood donors, and the second database involves the classification of image segments to identify diseased trees. In both databases, the RPDLomax model outperformed conventional link functions such as logit, probit, cauchit, loglog, and cloglog, showing the lowest fit metrics (WAIC, LOO, DIC, EAIC, and EBIC).

Finally, it is worth mentioning that all codes developed for this work can be found on the first author’s GitHub: https://github.com/leticiaferreiramurca/Msc.
